# Genome-Wide Identification and Expression Analysis of *Chitinase-like Genes* in *Petunia axillaris*

**DOI:** 10.3390/plants11091269

**Published:** 2022-05-09

**Authors:** Zhuoyi Liu, Wenfei Yu, Xiaowen Zhang, Jinfeng Huang, Wei Wang, Miao Miao, Li Hu, Chao Wan, Yuan Yuan, Binghua Wu, Meiling Lyu

**Affiliations:** 1 College of Horticulture, Fujian Agriculture and Forestry University, Fuzhou 350002, China; zhuoyi_liu@126.com (Z.L.); 1190371011@fafu.edu.cn (W.Y.); 3210330075@fafu.edu.cn (X.Z.); 1190371002@fafu.edu.cn (J.H.); mykuangwen@hotmail.com (W.W.); 2190514007@fafu.edu.cn (M.M.); 1200371004@fafu.edu.cn (L.H.); 1190371007@fafu.edu.cn (C.W.); yuanyuan@fafu.edu.cn (Y.Y.); binghua.wu@fafu.edu.cn (B.W.); 2 College of Horticulture, South China Agriculture University, Guangzhou 510642, China

**Keywords:** *chitinase-like genes*, evolution, gene expression, abiotic stress, *Petunia axillaris*

## Abstract

Chitinase (EC 3.2.1.14) is a kind of chitin-degrading glycosidase, which plays important roles in the abiotic and biotic defense of plants. In this study, we conducted whole-genome annotation, molecular evolution, and gene expression analyses on the *chitinase-like* (*CTL*) gene family members of *Petunia axillaris*. Thirty-three *Petunia axillaris* *chitinase-like genes* (*PaCTLs*) were identified from the latest Petunia genome database. According to the phylogenetic analyses, these genes were divided into GH18 and GH19 subgroups and further subdivided into five classes (Class I to Class V). Conserved motif arrangements indicated their functional relevance within each group. The expansion and homeology analyses showed that gene replication events played an important role in the evolution of *PaCTLs* and the increase of the GH18 subgroup members was the main reason for the expansion of the *PaCTL* gene family in the evolution progress. By qRT-PCR analysis, we found that most of the *PaCTLs* showed a very low expression level in the normal growing plants. But lots of *PaCTLs* showed upregulated expression profiles when the plants suffered different abiotic stress conditions. Among them, five *PaCTLs* responded to high temperature and exhibited significantly upregulate expression level. Correspondingly, many hormone responses, as well as biotic and abiotic stress elements were found in the promoters of *PaCTLs* by using cis-acting element analysis. These results provide a foundation for the exploration of *PaCTLs*’ function and enrich the evolutionary process of the *CTL* gene family.

## 1. Introduction

Chitinase is a glycoside hydrolase capable of hydrolyzing chitin. In plants, it is one of the pathogenesis-related genes (PRs) [1]. Although plants do not contain chitin, the expression activity of many chitinases was enhanced under the influence of abiotic and biotic stresses and exogenous hormones [2,3,4]. Experiments in recent years have proved that it also plays an important regulatory role in the growth and development of plants.

Based on previous research, chitinase-like proteins (CTLs) can be induced to product while the plant suffering with the pathogens, salt, drought, intracellular Ca^2+^, and freeze damage ect [5,6,7]. The *chitinase-like* *genes* (*CTLs*) cloned in *Mulberry badnavirus*, *Oryza sativa*, and *Arabidopsis thaliana* played important roles in the pest, disease, and environmental stress defense [8,9,10]. Furthermore, some plant species could grow better when introduced with exogenous chitinase during the vegetative growth process. The expression of a rice chitinase gene in transgenic banana enhances the resistance to black leaf streak disease [5]. The expression of rice chitinase gene in genetically engineered tomato confers enhanced resistance to *Fusarium wilt* and *Early blight* [6]. In addition, lots of *CTLs* have been characterized in plants in regulating their growth and developmental processes. Robinson et al. [11] showed that some chitinase were closely related to the maturation of grapes and had tissue or organ expression specificity. In carrots, the chitinase gene can participate in the control of the development of early embryos [12]. Jiao et al. [13] proved that the overexpression of *ZmCTL1* in maize enhanced mechanical stalk strength without affecting plant stature, senescence, or fertility. The AtCTL1 in *A. thaliana* negatively regulated ACO (1-aminocyclopropane-1-carboxylic acid oxidase) activity and ethylene response [14]. When one *CTL* in *Capsicum annuum*, *CaChiIV1*, is silenced, plants suffered weakened defense because root activity is reduced to 60% and susceptibility to salt stress is increased [1]. Transgenic tea could grow better and has enhanced resistance to *blister blight* through the overexpression of the Class I chitinase gene from potato [7]. Moreover, *CTLs* also have been proven to participate in the photosynthesis process [15].

In plants, *CTLs* are encoded by a multi-gene family. Recently, 16 and 43 *CTLs* were identified from *C. annuum* and *Solanum lycopersicum*, respectively [1,10]. Based on the amino acid sequence similarity and the presence or absence of a domain, they were divided into two glycosyl hydrolase subgroups (GH18 and GH19 subgroups). Kesari et al. [9] subdivided chitinase into six categories (Class I–VI), in which Class III and Class V belong to the GH18 subgroup, and the rest belong to the GH19 subgroup. Their research point is that the homology between Classes III and V is very low, and both classes have no similarity with the chitinase sequences of the GH19 subgroup. Besides, chitinase were divided into five categories (Class I–V) in some other reports based on amino acid sequence homology, 3D protein structure, substrate specificity, catalytic reaction mechanism, sensitivity to inhibitors, and other properties [1,8,10,16,17,18,19]. Among them, Class I and Class IV members have chitin-binding domain (CBD) and GH19 domain (the difference between them is that Class I also has a C-terminal conserved domain). The CBD structure is essential for chitinase because it can enhance resistance to abiotic and biotic stresses and promote plant growth and development. According to the research of Yamagami and Funatsu [20], the chitinase with CBD were three times more active against colloidal chitin than the other without CBD. Furthermore, the chitinase will have little change in the hydrolysis of ethylene glycol chitin if CBD was removed, but its activity on colloidal chitin will only be equivalent to 50% before removal. Moreover, the chitinase with CBD is more resistant to pathogens than the chitinase without CBD [21]. These results indicated that identifying chitinase and analyzing their structures are very important in exploring their function in growth regulation and stress response.

Petunia is an important model plant in the Solanaceae family. It is widely used in the study of flower molecular biology. The growth of petunia is affected by many biotic and abiotic stress factors, which led to a decline in their growth, quality, and ornamental nature. As a plant is unable to escape attacking biotic and abiotic factors by moving to a more favorable environment, it is necessary to possess efficient defense mechanisms to protect itself from disease [22]. However, at present, only a small amount of literature has been published on the genetic verification of petunia’s chitinase [23,24]. Moreover, no systematic investigation has been conducted on the *CTLs* family in petunia. In the current study, we identified 33 *Petunia axillaris* *chitinase-like genes* (*PaCTLs*) and performed an integrated analysis of their phylogeny, gene structures, motifs, and cis-acting elements of promoters to further understand their mechanisms. Subsequently, we characterized their expression profiles in different tissues under normal growth conditions and in seedlings under abiotic stress conditions. Our research provides a foundation for the further characterization of *PaCTLs* and enriches the evolutionary process of the *CTL* gene family.

## 2. Results

### 2.1. Identification of Putative *CTLs* in P. axillaris Genome

According to sequence alignment and functional domain analysis, we identified 33 *PaCTLs* in the petunia genome, which were distributed in 23 scaffolds (Table S1). The predicted results showed that their amino acid lengths vary greatly, ranging from 95 AA (Pa00167g00056.1) to 503 AA (Pa00180g00126.1). Among them, 26 *PaCTLs*, which comprised 24 secretory proteins and two mitochondrial proteins contained a signal peptide respectively. The remaining seven members (Pa00111g00047.1, Pa00836g00311.1, Pa00589g00225.1, Pa00585g00083.1, Pa00045g00450.1, Pa00347g00041.1, and Pa00347g00043.1) did not contain signal peptide. Furthermore, according to the predictions of three different sites, CELL02G0, BUSCA and WoLF PSORT (Table S2), all the GH19 proteins were located extracellular. However, the location of GH18 proteins were more complex, which could be found in the nucleus, chloroplasts, mitochondria, etc.

**Figure 1:**
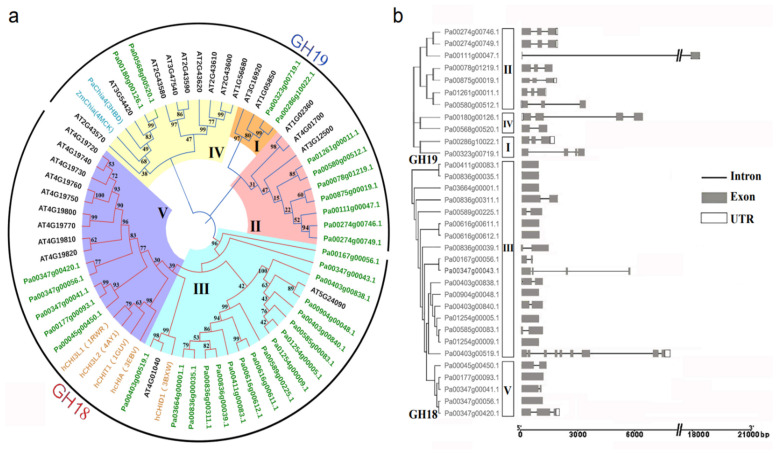
Classification and gene structure analyses of predicted *PaCTLs*. (**a**) Phylogenetic tree of the *CTLs* from *P. axillaris*, *A. thaliana*, *Z. mays*, *P. abies*, and *H. sapiens* (the names marked in green, black, dark cyan and orange respectively). The five classes (I–V) were assigned according to the ortholog structure and function published in other research report. (**b**) Schematic depictions of the exon-intron organizations of the *PaCTLs*.

We constructed a phylogenetic tree using the chitinase sequences of *A. thaliana*, *H. sapiens*, *P. abies*, and *Z. mays* to better understand the similarity, difference, and classification of PaCTL gene family members (Figure 1a). The result showed that all the 65 *CTLs* were classified into two subgroups, the GH18 and GH19. There were twenty-two (66.7%) and eleven (33.3%) numbers belonged to GH18 and GH19, respectively. According to the sequence similarity and previous research [1,10,19], the CTL numbers were further subdivided them into five classes (Class I–V). Among them, class III and class V belonged to the GH18 subgroup including seventeen and five numbers, respectively. Class I, II, and IV belonged to the GH19 subgroup and comprised of two, seven, and two numbers, respectively.

### 2.2. Analysis of the Gene Structure and Conserved Motifs of the *PaCTLs*

Intron–exon structure analysis was performed to further study the structural diversity of *PaCTLs* (Figure 1b). The result showed that all of the *PaCTLs* in GH19 subgroup contained introns. In contrast, only twelve members (54.5%) in GH18 subgroup contained introns, and the member *Pa00403g00519.1* contained the most introns (8 introns). Among them, *Pa00111g00047.1* had the longest intron sequence. Ten members of the 33 *PaCTLs* (30.3%) did not contain introns, 11 members (33.3%) contained one intron, eight members (24.2%) contained two introns, and three members (9%) contained three introns. 

**Figure 2:**
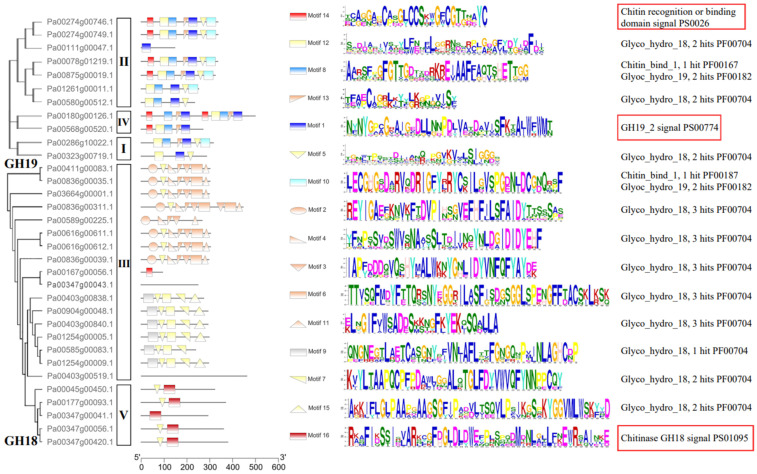
Domain architecture of predicted *PaCTLs*. The motifs indicated by red boxes were the typical motifs of the GH18 and GH19 subgroups.

The conserved motifs were investigated to analyze the diversification of PaCTL proteins (Figure 2, Figures S1 and S2). We analyzed 16 putative motif sequences that could correspond to the different conservative structures of the GH group by Pfam search in NCBI. The members of a certain class exhibited higher identity. All the members of Classes I, II, and IV belonged to the GH19 subgroup contained the GH19_2 signal motif. Four members of Class II and both members of Class IV contained the chitin recognition or binding domain signal motif. Almost all of the *PaCTLs* in GH18 subgroup contained the Glyco_hydro_18 motif except two members (Pa00167g00056.1 and Pa00347g00043.1), and all the five members belonged to Class V contained chitinase GH18 signal motif. Though the GH18 conserved domain of two members (Pa00403g00519.1 and Pa00347g00043.1) did not detect by MEME online service website, it could be searched through the CD-search function in the NCBI website.

### 2.3. The Evolution Events of the CTL Gene Family in P. axillaris

**Figure 3:**
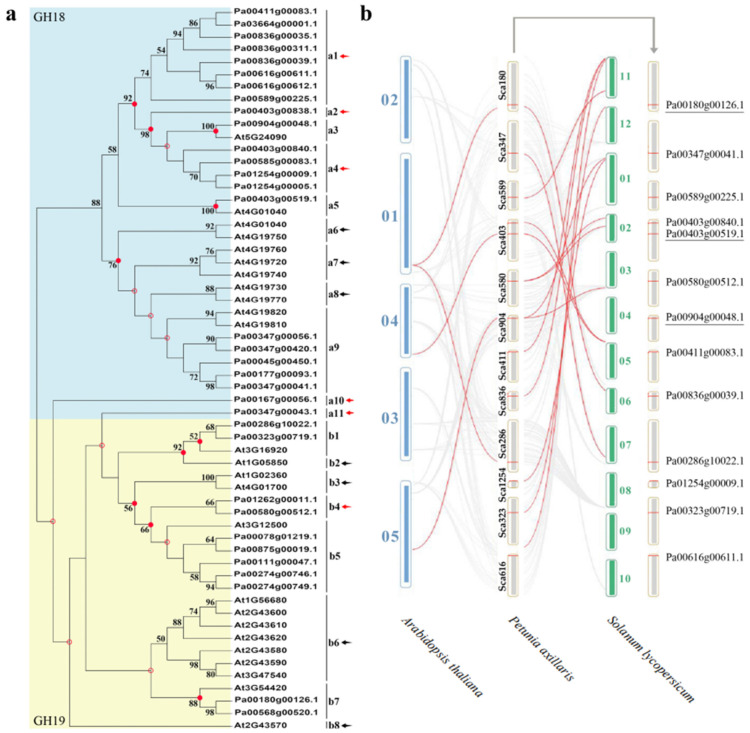
Phylogenetic tree construction and homeology analysis. (**a**) The phylogenetic tree of *CTLs* from *P. axillaris* and *A. thaliana*. Numbers on branches indicate the bootstrap percentage values calculated from 1000 replicates, and only values higher than 50% are shown. The nodes that represent the most recent common ancestral genes before the *P. axillaris* and *A. thaliana* split are indicated by red dots (bootstrap support > 50%) and hollow dots (bootstrap support < 50%). Clades that contain only *P. axillaris* and *A. thaliana* *CTLs* are indicated by red and black arrows, respectively. (**b**) The homeology analysis of *CTLs* from petunia, *Arabidopsis* and tomato genomes. The gray lines in the background indicate the homologous relationship within *P. axillaris* and the other two plant genomes, while the red lines highlight the members of the *CTL* gene family that have a homologous relationship between two genomes.

**Figure 4:**
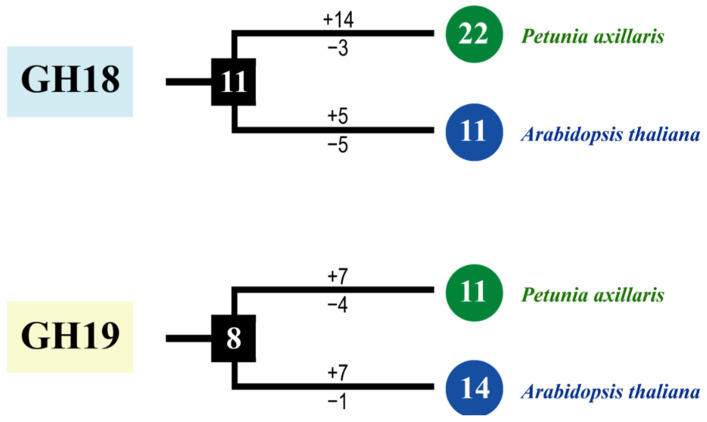
The copy number changes of *P. axillaris* and *A. thaliana CTLs*. Numbers in ellipses and rectangles represent the numbers of *CTLs* in extant and ancestral species, respectively. Numbers on branches with plus and minus symbols represent the numbers of gene gains and losses, respectively.

As shown in Figure 3a, 33 *PaCTLs* and 25 *Arabidopsis* *CTLs* members were grouped into two major groups, GH18 and GH19 subfamilies. According to the phylogenetic relationships, we identified the nodes that lead to *A. thaliana*–specific and *P. axillaris*–specific branches, which represented the most recent common ancestral genes before they split. The results showed that there were at least 19 *CTLs* ancestor genes between the two species. There are 6 branches contained only *PaCTLs* (red arrow) and 7 branches contained only *Arabidopsis* *CTLs* (black arrow), indicating that these branches may have occurred gene losses during the evolutionary process. Meanwhile, we calculated the obtained or lost of *CTLs* in *A. thaliana* and *P. axillaris* during the evolution progress. In the GH18 subfamily, there were at least 11 common *CTLs* ancestor genes between petunia and *Arabidopsis* (Figure 4). After split, petunia gained 14 and lost 3 members, and *Arabidopsis* gained 5 and lost 5 members, resulting in 22 and 11 GH18 subfamily members, respectively. Apparently, petunias gained much more new members than *Arabidopsis*, which has led to the rapid expansion of the GH18 subfamily in petunia. In the GH19 subfamily, there were at least 8 common *CTLs* ancestor genes between petunia and *Arabidopsis* (Figure 4). During the evolution process, petunia gained 7 and lost 4 *CTLs* members, *Arabidopsis* gained 7 and lost 1 *CTLs* members, which resulted in more GH19 subfamily *CTLs* members in *Arabidopsis* than that in petunia.

Homology analysis of *CTL* gene family members in *A. thaliana*, *P. axillaris* and *S. lycopersicum* indicated that there were homologous relationships among them. As shown in Figure 3b, lots of *CTLs* in the *A. thaliana* and *S. lycopersicum* genomes had homologous relationship with *PaCTLs*. Furthermore, among the 33 *PaCTLs*, four members (*Pa00180g00126.1*, *Pa00904g00048.1*, *Pa00403g00519.1* and *Pa00286g10022.1*) were homologous with three *CTLs* in *A. thaliana* genomes. There were 12 *PaCTLs* members (*Pa00904g00048.1*, *Pa00589g00225.1*, *Pa00580g00512.1*, *Pa00180g00126.1*, *Pa00836g00039.1*, *Pa00403g00519.1*, *Pa00403g00840.1*, *Pa00411g00083.1*, *Pa00323g00719.1*, *Pa00347g00041.1*, *Pa01254g00009.1*, *Pa00616g00611.1*) were homologous with 10 members in *S. lycopersicum* genome. Three *PaCTLs* members (*Pa00180g00126.1*, *Pa00904g00048.1* and *Pa00403g00519.1*) were homologous with *CTLs* both in *A. thaliana* and in *S. lycopersicum* genomes.

To explore the relationship of the orthologous gene pairs of *CTLs* between *S. lycopersicum* and *P. axillaris*, the nonsynonymous substitution rates (*Ka*), synonymous substitution rates (*Ks*) and *Ka*/*Ks* were calculated. According to the result, all the *Ka*/*Ks* ratios were less than 0.5, which indicated that purifying selection was the dominant force driving the evolution of *CTLs* between the two species (Table S3). Furthermore, we constructed the phylogenetic tree using the *CTLs* of five Solanaceous species (*P. axillaris*, *N. tabacum*, *S. lycopersicum*, *S. tuberosum*, *and C. annuum*) and *A. thaliana* (Figure S3). The statistics on the member numbers of the different classes (I–V) of the six species (Table S4) showed that the *CTLs* in *Solanaceous* plants belong to Class III increased remarkably compared with those in *A. thaliana*. Moreover, the *CTLs* of *S. lycopersicum* and *S. tuberosum* in Class II increased rapidly and were all 11 more than in the Class II of *A. thaliana*. These finding indicated that the increase of the GH18 subgroup members might be the main cause for the expansion of *CTL* gene family in *P. axillaris*, *N. tabacum*, and *C. annuum*, while the increase of *CTLs* in Class III and Class II might be the main cause for the expansion of *CTL* gene family in *S. lycopersicum* and *S. tuberosum*.

### 2.4. Profiling of *PaCTLs* Expression in Different Tissues

**Figure 5:**
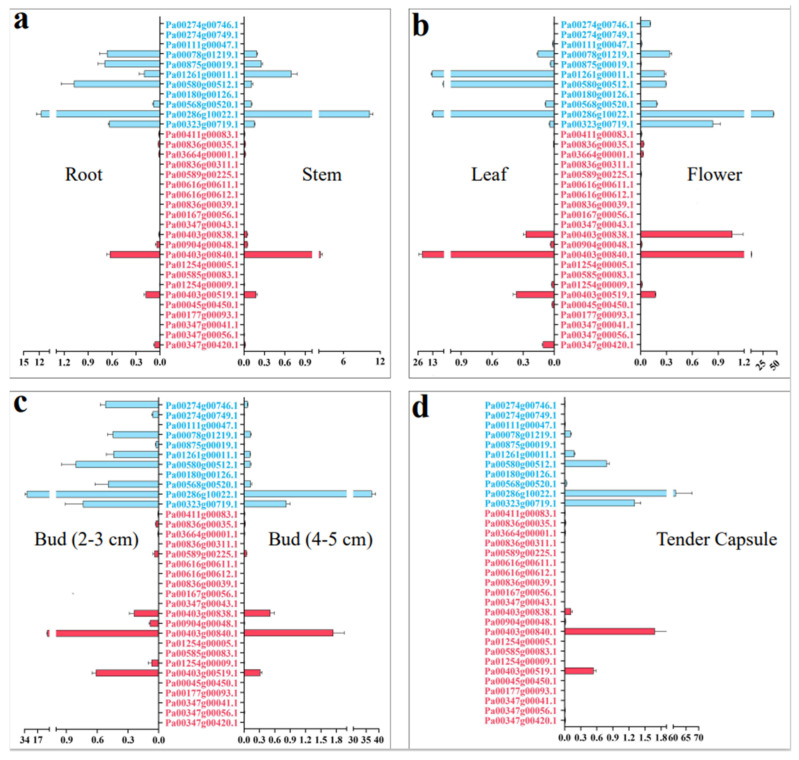
Relative expression of the *CTLs* family members in the different tissues of *P. axillaris*. (**a**) Expression pattern in root and stem. (**b**) Expression pattern in leaf and open flower. (**c**) Expression pattern in 2–3 cm and 4–5 cm flower. (**d**) Expression pattern in tender capsule tissues. The genes marked in blue were the members of the GH19 subgroup, and the genes marked in red were the members of the GH18 subgroup.

We detected the relative expression of *PaCTL* gene family members in seven tissues (roots, stems, leaves, 2–3 cm flower buds, 4–5 cm flower buds, open flowers, and tender capsule) by using qRT-PCR to further elucidate their expression characteristics in various vegetative and reproductive tissues of the normal growing plants (Figure 5). The results presented that the expression divergence between the *PaCTLs* of GH18 and GH19 sub group was quite obvious. Among the 11 *PaCTLs* in the GH19 subgroup, two genes (*Pa00111g00047.1* and *Pa00180g00126.1*) were not expressed in all the seven tissues, and other nine genes (81.8%) could express in at least one tissue of the plant. It is worth mentioning that one gene, *Pa00286g10022.1*, had a higher expression level in reproduction tissue, and its expression level showed a gradual increase along with the reproductive development process. Among the *PaCTLs* in the GH18 subgroup, 14 members (66.7%) did not express in any of the seven tissues. One gene (*Pa00403g00840.1*) had a higher expression level in leaves. Taken together, most of the *PaCTLs* showed a very low expression level in normal growing plants.

The *Ka*, *Ks*, and *Ka*/*Ks* of eight *PaCTLs* paralog pairs were calculated to explore the relationship among the *Ka*, *Ks*, and the expression pattern of *PaCTLs* (Table S5). The result showed that the *Ka*/*Ks* value of one pair (*Pa00274g00749.1*/*Pa00274g00746.1*) was greater than 1, which indicated that they suffered a positive selection in the evolution process. The *Ka*/*Ks* values of the remaining seven pairs were all less than 1, which indicated that these pairs suffered purifying selection during the evolution progress. Genes with a higher degree of similarity tend to have similar expression characteristics. We verified whether the same event could be observed in *PaCTLs* by investigating the expression characteristics of the eight paralog pairs. Combined with the qRT-PCR analysis of the *PaCTLs* in different tissues (Figure 5), we found that these paralog genes could be divided into two kinds of expression patterns. In the first mode (mode I in Table S5), the paralog genes showed selective expression in different tissues. For example, one gene was expressed in the flower, and the other was not. A pair of paralog genes (*Pa00274g00749.1*/*Pa00274g00746.1*), which suffered a positive selection, falls into this category. In the second mode (mode II in Table S5), both of the paralog genes showed very low expression levels. Seven paralog pairs (*Pa00836g00035.1*/*Pa00411g00083.1*, *Pa00836g00035.1*/*Pa00836g00311.1*, *Pa00411g00083.1*/*Pa03664g00001.1*, *Pa00411g00083.1*/*Pa00836g00311.1*, *Pa00616g00611.1*/*Pa00616g00612.1 Pa00836g00035.1*/*Pa03664g00001.1*, and *Pa03664g00001.1*/*Pa00836g00311.1*) showed this expression pattern.

### 2.5. Expression Patterns of *PaCTLs* under Abiotic Stress and Hormone Treatment

**Figure 6:**
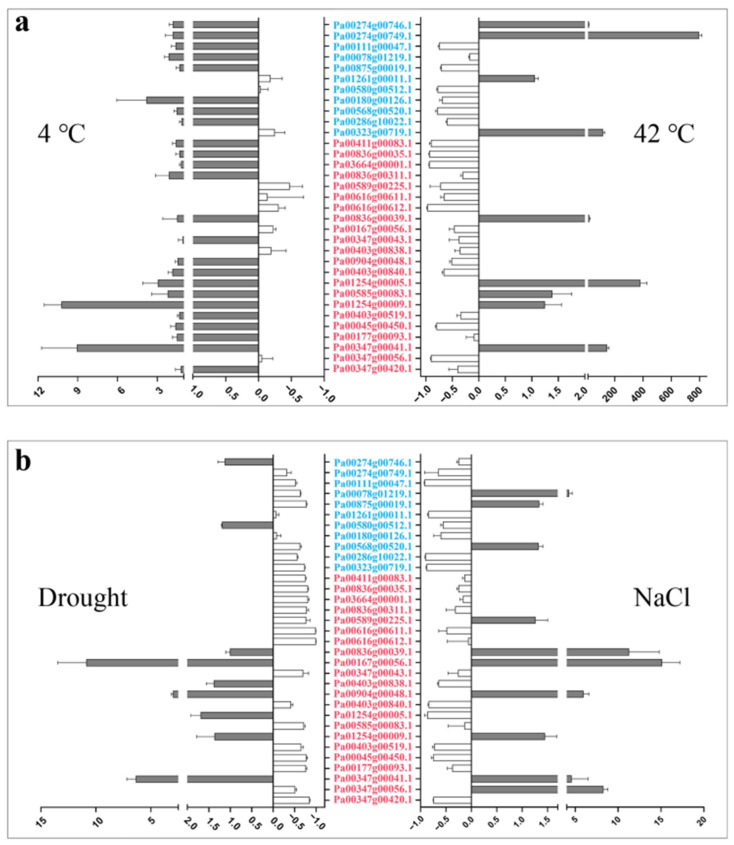
Relative expression patterns of *PaCTLs* under abiotic stress conditions. (**a**) Relative expression patterns of the *PaCTLs* under low and high temperature stress. (**b**) Relative expression patterns of members of the *PaCTLs* under drought and salt stress. The abscissa value greater than 0 indicated the expression of *PaCTL* was up-regulated; The abscissa value less than 0 indicated the expression of *PaCTL* was down-regulated. The genes marked in blue were the members of the GH19 subgroup, and the genes marked in red were the members of the GH18 subgroup.

We detected the expression of the 33 *PaCTLs* under low-temperature (4 °C), high-temperature (42 °C), drought, and salt stress environments in the seedlings to investigate their response to the stress environment (Figure 6 and Table S6). The result showed that, when the seedlings were subjected to a low-temperature environment, the expression levels of two members (*Pa00347g00041.1* and *Pa01254g00009.1*) were remarkably increased compared with the control, and two other members (*Pa00180g00126.1* and *Pa01254g00005.1*) also showed slightly up-regulated expression. When the seedlings were subjected to high-temperature environment, six members (*Pa00323g00719.1*, *Pa00274g00746.1*, *Pa00274g00749.1*, *Pa00836g00039.1*, *Pa00347g00041.1*, and *Pa01254g00005.1*) were expressed much higher than those in the control and 13 members (*Pa00111g00047.1*, *Pa00875g00019.1*, *Pa00580g00512.1*, *Pa00180g00126.1*, *Pa00568g00520.1*, *Pa03664g00001.1*, *Pa00836g00035.1*, *Pa00411g00083.1*, *Pa00616g00612.1*, *Pa00589g00225.1*, *Pa00403g00840.1*, *Pa00347g00056.1*, and *Pa00045g00450.1*) were remarkably downregulated. In the drought environment, the relative expression levels of three members (*Pa00167g00056.1*, *Pa00347g00041.1*, and *Pa00904g00048.1*) were upregulated. Thirteen members (*Pa00875g00019.1*, *Pa00323g00719.1*, *Pa03664g00001.1*, *Pa00836g00035.1*, *Pa00411g00083.1*, *Pa00836g00311.1*, *Pa00616g00611.1*, *Pa00616g00612.1*, *Pa00589g00225.1*, *Pa00585g00083.1*, *Pa00347g00420.1*, *Pa00045g00450.1*, and *Pa00177g00093.1*) had more than three times lower expression level than those in control. In addition, six members (*Pa00078g01219.1*, *Pa00347g00041.1*, *Pa00347g00056.1*, *Pa00836g00039.1*, *Pa00904g00048.1*, and *Pa00347g00056.1*) could respond to salt stress, and their expression level showed a certain increase. Nine members (*Pa00111g00047.1*, *Pa01261g00011.1*, *Pa00286g10022.1*, *Pa00323g00719.1*, *Pa00403g00840.1*, *Pa01254g00005.1*, *Pa00403g00519.1*, *Pa00347g00420.1*, and *Pa00045g00450.1*) were downregulated during salt stress. Taken together, the results showed that low temperature had the least effect on the expression of *PaCTLs*. The member *Pa00347g00041.1* was upregulated in all of the four stress treatments. The upregulation of *Pa00167g00056.1*, *Pa00836g00039.1*, *Pa01254g00005.1*, and *Pa00904g00048.1* could be observed in two different stress treatments. Furthermore, the expression level of most of the GH19 subgroup members was slightly changed except for two members (*Pa00274g00749.1* and *Pa00323g00719.1*) which were considerably upregulated in response to high-temperature stress. The expression characteristics of GH18 subgroup members were quite divergent, but an obvious upregulation can be found in all the four stress treatments.

**Figure 7:**
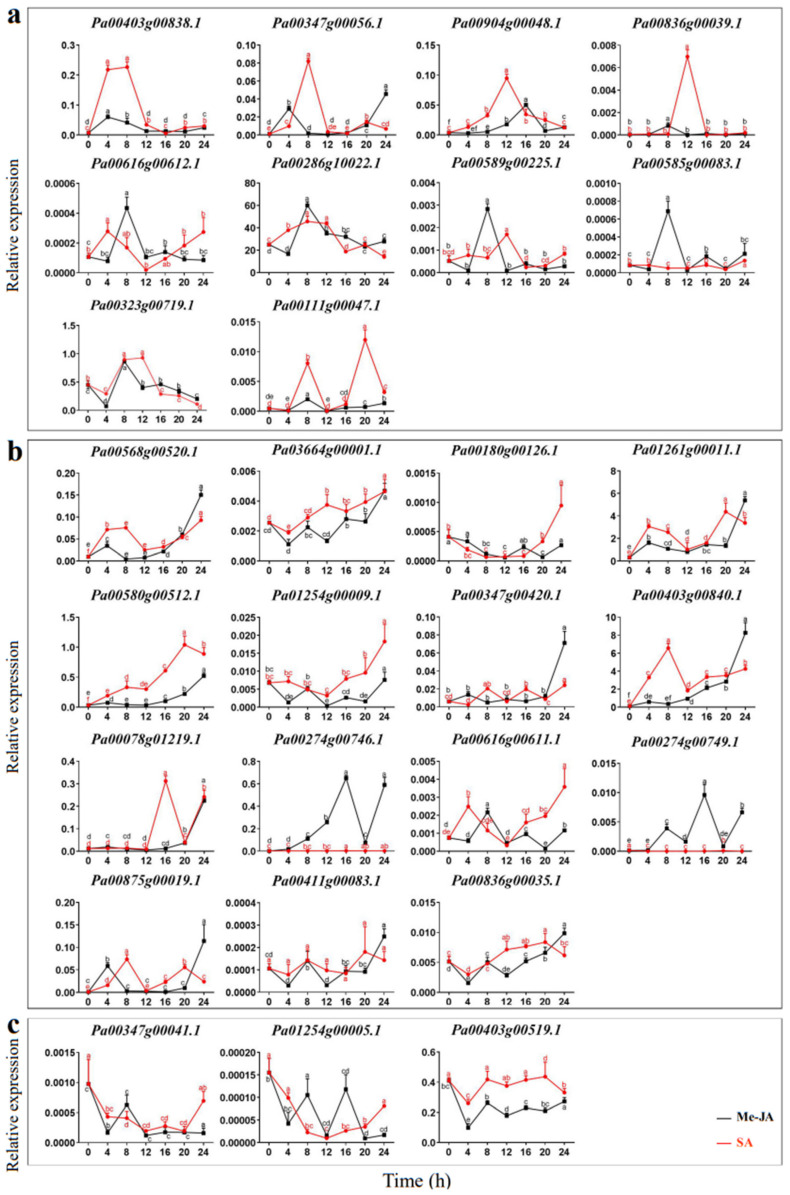
Relative expression patterns of *PaCTLs* under the treatment of exogenous hormones. (**a**) The expression of *PaCTLs* showed a fluctuating recovery under the influence of exogenous hormones. (**b**) The expression of *PaCTLs* showed a fluctuating up-regulated under the influence of exogenous hormones. (**c**) The expression of *PaCTLs* showed a fluctuating down-regulated under the influence of exogenous hormones. Significant difference between group with variable letters above the line or bar are verified using two-way ANOVA and multiple *t*-test (*p* < 0.05) implemented in the Prism software version 8.

The expression patterns of *PaCTLs* were detected by qRT-PCR in the plants treated with Me-JA solution and SA solution after 0, 4, 8, 12, 16, 20, and 24 h respectively. As shown in Figure 7, the expression level could be classified into three kinds of response types, Including volatility recovery, volatility up, and volatility down. In the first type (Figure 7a), after being stimulated by exogenous Me-JA (methyl jasmonate) or SA (salicylic acid), *PaCTLs* formed a small peak of expression level, and then return to the expression level before treatment. There were 10 members (*Pa00616g00612.1*, *Pa00286g10022.1*, *Pa00589g00225.1*, *Pa00585g00083.1*, *Pa00323g00719.1*, *Pa00111g00047.1*, *Pa00403g00838.1*, *Pa00347g00056.1*, *Pa00904g00048.1* and *Pa00836g00039.1*) belonged to this type. In the second type (Figure 7b), *PaCTLs* showed a fluctuating upward trend after being stimulated by exogenous hormones, and their relative expression level after 24 h were still higher than that before treatment. There were 15 members in total (*Pa00568g00520.1*, *Pa03664g00001.1*, *Pa00180g00126.1*, *Pa01261g00011.1*, *Pa00580h00512.1*, *Pa01254g00009.1*, *Pa00347g00420.1*, *Pa00403g00840.1*, *Pa00078g01219.1*, *Pa00274g00746.1*, *Pa00616g00611.1*, *Pa00274g00749.1*, *Pa00875g00019.1*, *Pa00411g00083.1* and *Pa00836g00035.1*) falls into this category. In the third type (Figure 7c), the stimulation of exogenous hormones caused a decrease of the relative expression levels of *PaCTLs* members, but they basically returned to the pre-treatment levels after 24 h. Three members (*Pa00347g00041.1*, *Pa01254g00005.1* and *Pa00403g00519.1*) belonged to this type. Five other members (*Pa00836g00311.1*, *Pa00167g00056.1*, *Pa00347g00043.1*, *Pa00045g00450.1* and *Pa00177g00093.1*) had little change in their relative expression level after being stimulated by Me-JA and SA.

### 2.6. Cis-Acting Element Analysis of Promoter Region

**Figure 8:**
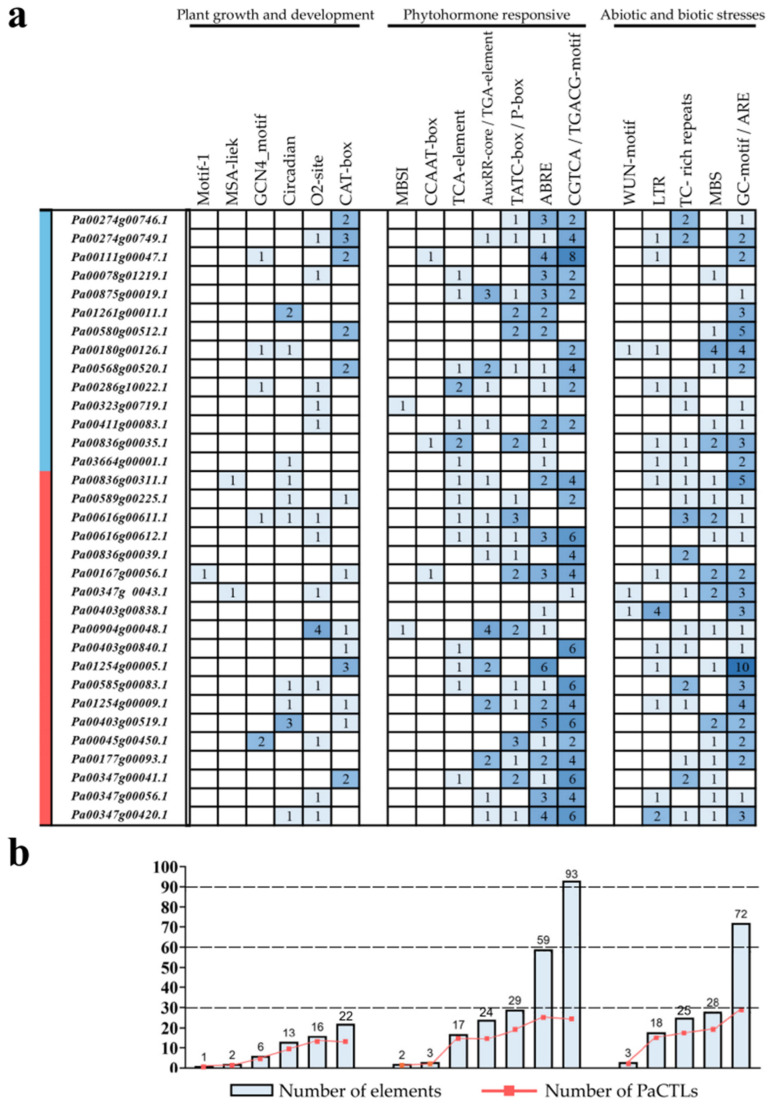
Cis-acting elements analysis in the promoter of *PaCTL* gene family members. (**a**) Number of each cis-acting element in the promoter region (2 kb upstream of the translation start site) of *PaCTLs*. The genes marked in blue region were the members of the GH19 subgroup, and the genes marked in red region were the members of the GH18 subgroup. (**b**) Statistics for the total number of *PaCTLs*, including the corresponding cis-acting elements (red dot) and the total number of cis-acting elements in *PaCTL* gene family (blue box). Based on the functional annotation, the cis-acting elements were classified into three major classes: plant growth and development-, phytohormone responsive-, or abiotic and biotic stresses-related cis-acting elements.

The 2-kb upstream regions of the translation start site of the *PaCTLs* were analyzed to further investigate the possible involvement of cis-acting elements in the stimulation of defense-related genes. Statistical analysis revealed that multiple cis-acting elements conferring responsiveness to biotic and abiotic stresses and plant hormones were found in the promoters (Figure 8). The promoters of 15 *PaCTLs* contained defense and stress response elements (TC-rich repeats). Twenty promoters contained drought-inducing response elements (MBS), and 14 promoters included cryo-responsive elements (LTR). Anaerobic inducing element (ARE) widely existed and was missing in only three promoters (the promoters of *Pa00286g10022.1*, *Pa00347g00041.1*, and *Pa00836g00039.1*). Moreover, more than 5 AREs existed in the promoters of *Pa00580g00512.1*, *Pa00836g00311.1*, and *Pa01254g00005.1*. Nine promoters contained circadian response elements. A total of 26, 23, 25, 14, and 14 promoters contained abscisic acid response element (ABRE), jasmonic acid response element (CGTCA-motif), salicylic acid response element (TCA-element), gibberellin response element (TATC-box), and auxin response element (AuxRR-core), respectively. Many promoters contained multiple cis-acting elements. In addition, one promoter (the promoter of *Pa00167g00056.1*) contained the root-specific cis-element (motif I), two promoters (the promoters of *Pa00403g00838.1* and *Pa00374g0043.1*) contained the wound response element (WUN-motif), one promoter (the promoter of *Pa00323g00719.1*) contained the flavonoid biosynthesis gene regulatory element (MBSI), and two promoters (the promoters of *Pa00836g00311.1* and *Pa00374g0043.1*) contained cell cycle regulatory element (MSA-like).

### 2.7. Subcellular Localization of PaCTL Proteins

**Figure 9:**
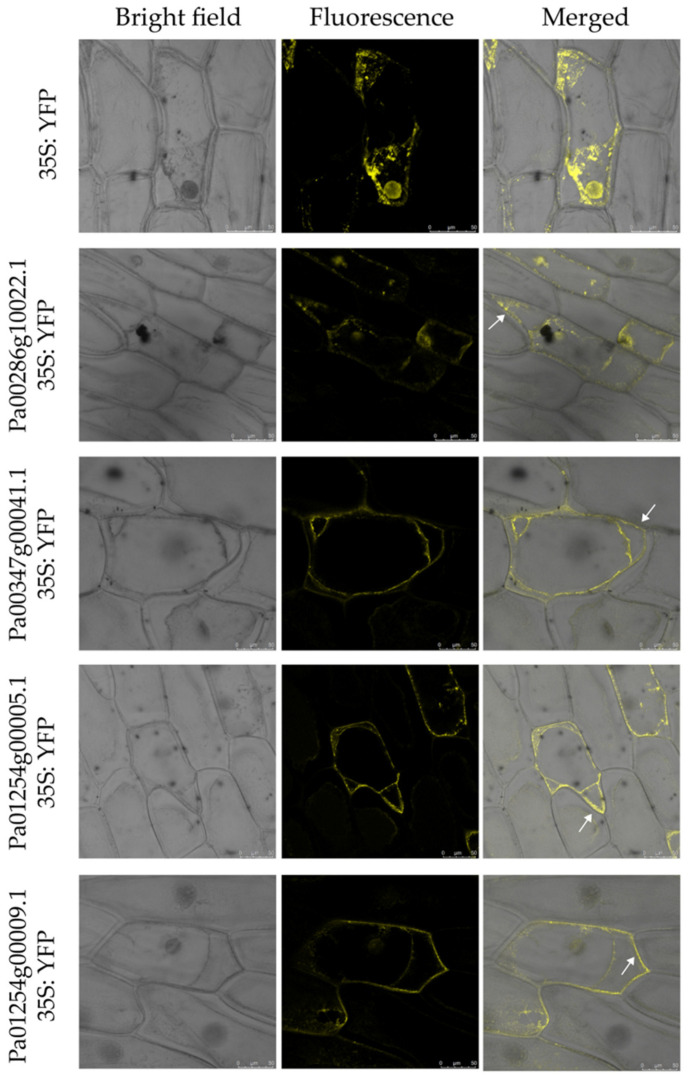
Subcellular localization of four *PaCTLs*-yellow fluorescent protein (YFP) fusion proteins in onion epidermal cells. The middle row show fluorescence images of plasmolyzed transgenic cells. The fluorescence signal can be observed in the cell wall (white arrows). The left column are bright-field images of the corresponding onion epidermal cells. Scale bars = 50 µm.

In this study, the three programs (CELL02G0, BUSCA and WoLF PSORT) were used to predict the subcellular location of eukaryotic proteins (Table S2). The result showed that these proteins were mainly located in extracellular, which indicated that these proteins might be secreted proteins. In order to examine the accuracy of the prediction results, we selected four PaCTL proteins (Pa00286g10022.1, Pa00347g00041.1, Pa01254g00005.1 and Pa01254g00009.1), which had a higher expression level in flower organ and stress environment to construct subcellular localization fusion expression vectors. After transient expression of the fusions in onion epidermis cells, the yellow fluorescent protein (YFP) signal were observed by confocal microscopy. Observations indicated that the fluorescent signals of the four proteins were ubiquitously distributed in the onion epidermal cells. To determine if these proteins could be localized on the cell wall, we performed a plasmolysis experiment by treatment with 0.3 g·mL^−1^ sucrose. After plasmolysis, the fluorescent signals could be observed in the cell wall (Figure 9). The results above indicated that the four PaCTL proteins were secreted proteins and could localize on the cell wall.

## 3. Discussion

### 3.1. Evolution Analysis of the *PaCTLs*

Carbohydrate-active enzymes often exhibit a modular structure containing non-catalytic carbohydrate-binding modules, which enhance enzymatic activity by positioning the substrate closer to the catalytic domain [21]. Similarly, One CTL protein has multiple CBD domains, which may be closely related to the efficiency of protein binding to chitin [25,26]. Yamagami and Funatsu [20] demonstrated that the *CTLs* that possessed a CBD domain had stronger hydrolytic activity in rye plants than those without a CBD domain. According to this study, six *PaCTLs* (*Pa00274g00746.1*, *Pa00274g00749.1*, *Pa00180g00126.1*, *Pa00568g00520.1*, *Pa00078g01219.1*, and *Pa00875g00019.1*) in the GH19 subgroup and one member (*Pa00167g00056.1*) in the GH18 subgroup have one or two CBD structures (Figure 2, Figures S1 and S2), which may be able to recognize and respond to chitin quickly. Here, we hypothesized that these seven *PaCTLs* might have stronger hydrolase activity, but further experiments are needed to verify these inferences.

Gene duplication and loss play an important role in the evolution of novel functions and for shaping an organism’s gene content [2]. In this study, we found that four pairs of *CTLs* in the genomes of *P. axillaris* and *A. thaliana* had a homology relationship (Figure 3b). Eleven additional pairs of *CTLs* collinearly occurred in the *P. axillaris* and *S. lycopersicum* genomes with the continuous evolution of the species. These findings might prove that the *CTLs* will continue to evolve into new genes while still functional for various reasons, such as individual needs or environmental factors, and playing a corresponding role in adapting to growth. The catalytic domain of the GH18 subgroup members has an (α/β) eight-barrel folded structure, which has been demonstrated by the structural analysis of melamine [27]. The catalytic domain of the GH19 subgroup members has a high α-helix component [28,29]. Moreover, GH18 and GH19 subgroup chitinases use two different hydrolysis mechanisms in plants. The chitinase of the GH18 subgroup uses a retention mechanism, whereas the chitinase of the GH19 subgroup catalyzes the overall transformation of the hetero-oligosaccharide configuration [30]. Therefore, GH18 and GH19 chitinases may have different evolutionary origins and functions [31]. According to the result in this research, the *CTLs* of the GH18 subgroup in Solanaceae plants increased much faster than those in *A. thaliana* after splitting (Table S4). Thus, the increase of GH18 subgroup members was the important reason for the expansion of the CTL gene family in Solanaceae plants. For *P. axillaris*, the *PaCTLs* of the GH18 subgroup increased rapidly after splitting with *A. thaliana* (Figure 4), while the *PaCTLs* of the GH19 decreased slightly. Thus, the increase in GH18 subgroup members was the main reason for the expansion of the *PaCTLs*. Furthermore, when investigated the expression characterization, we found that 81.8% *PaCTLs* of the GH19 subgroup can express in the plant, while 66.7% *PaCTLs* of the GH18 subgroup did not express in any of the seven tissues. This means that the *PaCTLs* of GH18 and GH19 subgroup may have functional differentiation during evolution. We hypothesized that the faster expansion of genes in the GH18 subgroup may provide new raw materials for the new functionalization or sub-functionalization of *CTLs*. If this conjecture is true, then the evolutionary selection of GH18 subgroup members in the Solanaceae plants may lead the repeat genes to form new specific expression patterns or functions.

### 3.2. Functional Diversity of PaCTL Gene Family Members

Chitinases are widely found in various organisms, such as plants, bacteria, fungi, viruses, arthropods and humans [32,33]. Although plants do not contain chitin, several *CTLs* have been shown to play important roles in their growth and development processes [13,14,15]. In our study, 11 *PaCTLs* had higher expression levels in one or more tissues of normally grown plants. For example, the expression of *Pa01261g00011.1*, *Pa00580g00512.1*, *Pa00286g10022.1*, and *Pa00403g00840.1* were localized in the leaves, and *Pa00286g10022.1* obviously increased during the progress of flower organ development ect. These findings might suggest that the presence of *PaCTLs* facilitated the growth or function of different organs. According to the previous research results of other scholars, plant chitinase played important roles in resisting fungi and bacteria and could also play a certain role in abiotic stress [34,35]. We detected the expression of the *PaCTLs* in low temperature (4 °C), high temperature (42 °C), drought, and salt conditions (Figure 6). We found that some genes could participate in different stress treatments. This result is similar to the previous studies in populus and soybean [36,37]. The *Pa00347g00041.1* gene was highly expressed in all four stress treatments conditions, while its relative expression under normal growth conditions was extremely low (Figure 5 and Figure 6; Table S6). This difference may indicate that this gene could be activated in respond to multiple abiotic stresses. Furthermore, others also could function when plants were subjected to abiotic stress, different genes selectively respond to one or several kinds of stresses. Such as *Pa00167g00056.1*, *Pa00836g00039.1*, *Pa01254g00005.1*, and *Pa00904g00048.1* were highly expressed in two to three stress treatment conditions. In addition, the divergent expression characteristics between GH18 and GH19 subgroup members were very obvious. Expression divergence is generally believed as the first step in functional divergence. Thus, we speculated that they may conduct divergent function in the growth of the *P. axillaris* plant. As a signaling molecule, Me-JA and SA are widely involved in plant stress resistance in biological and abiotic stresses, such as pests, UV-B, drought, high temperature and other stresses [38]. Under the condition of adversity stress, the content of endogenous JA (jasmonic acid) and Me-JA increases, which activates the self-defense system, induces the expression of related defense genes, synthesizes defense substances, and enhances the stress resistance of plants. In here, we found that nearly half of *PaCTLs* (45.4%) could up-regulated (Figure 7) when the plants suffered Me-JA and SA treatment, which further indicated that *PaCTLs* played an important role in the process of plants being subjected to environmental stress. It is worth mentioning that among the 33 *PaCTLs* members, *Pa00347g00043.1* was neither expressed under normal growth conditions nor under stress conditions (Figure 4 and Figure 5 and Table S6). Although this gene share similar chitinase conserved domains, it may not have biological functions, or may be pseudogene. Many chitinases have been reported to be localized on the plant cell wall, so it is speculated that the cell wall may be an important location for its action [39]. The subcellular localization results in this study (Figure 9) also validated this inference. However, our data is still limited. Further studies are urgently needed to determine whether the *PaCTLs* can play roles in responding to other abiotic and biotic stresses and how they function in petunia growth and development.

Cis-regulatory modules in promoters lead to specific shifts in the expression patterns and functions of genes. In our analysis, many hormone responses, as well as biotic and abiotic stress elements, were detected in the promoter of *PaCTLs* (Figure 8). For example, the expression levels of five genes (*Pa00347g00041.1*, *Pa01254g00009.1*, *Pa00180g00126.1*, and *Pa01254g00005.1*) were upregulated after low-temperature stress (4 °C). Among them, three genes had low-temperature response cis-elements in their promoter regions. Three promoters of *PaCTLs* (*Pa00347g00041.1*, *Pa01254g00005.1*, and *Pa00904g00048.1*) contained drought-induced cis-elements. Consistent with this, these three genes showed upregulated expression when the plants suffered drought stress. In addition, two genes also had increased expression under stress conditions, while their promoters did not contain corresponding cis-elements. The reason may be that the cis-element does not exist within the length of the detected 2-kb. Plant hormones and signal substances, such as Me-JA, SA, and abscisic acid, are involved in the operation of various stress signaling pathways [40,41,42,43]. A total of 26, 23, 25, 14, and 14 promoters of *PaCTLs* contained ABRE, CGTCA-motif, TCA-element, TATC-box, and AuxRR-core elements, respectively. Under the influence of exogenous Me-JA and SA hormones, *PaCTLs* showed three response types (Figure 7). Among the members of GH19 subfamily, 7 members could be affected by Me-JA hormones and 5 members were up-regulated when suffered SA hormones treatment. Among the members of GH18 subfamily, 4 members could be affected by Me-JA hormone and 5 members were up-regulated when suffered SA hormones treatment. These cases also have been found in the *CTL* genes of *S. lycopersicum* and *C. annuum* [1,10], possibly because of the functional consistency, enhancement, or weakening among homologous genes between species. According to the result, we speculated that the *PaCTLs* may also play important roles in various plant hormone signaling pathways. The specific response mode and triggering process need further research.

## 4. Materials and Methods

### 4.1. Plant Materials and Treatment

*P. axillaris* were provided by Dr. Hajirezaeiwere of the Leibniz Institute for Plant Genetics and Crop Research in Germany and cultivated in the experimental greenhouse of Fujian Agriculture & Forestry University. The growth conditions were 14 h light/10 h dark, 24 °C, and 70% humidity. 

The tissue samples under abiotic stress environment were obtained as follows: *P. axillaris* seeds were sterilized with 70% alcohol for 30 s in a clean bench. Then, the seeds were rinsed with sterile water thrice, sterilized with 2% sodium hypochlorite for 5 min, and rinsed with sterile water again for three times. Afterward, we sowed the seeds in Murashige and Skoog liquid medium. The seeds grew in 23 ± 1 °C under 14 h light/10 h dark environment until two leaves grow. Subsequently, the *P. axillaris* seedlings were subjected to low-temperature and high-temperature stress experiments in 4 ± 1 °C and 42 ± 1 °C, respectively. The seedlings were also placed in 150 mM NaCl for 24 h for the salt stress test. Furthermore, the seedlings were placed on folds of tissue paper at 23 ± 1 °C in 14 h light/10 h dark environment for drought stress experiments. The control was placed in a normal growth environment without treatment.

The tissue samples with hormone treatment were obtained as follows: The 50 µM Me-JA solution and 50 µM SA solution were prepared with 1 L volume, and then put them into the watering pot for using respectively. Petunia seedlings growing for 4 weeks (undifferentiated reproductive organs) were divided into two groups with 15 plants in each. One group was sprayed using the 1 L Me-JA solution, the other group were sprayed using the 1 L SA solution. The leaf materials before spraying were collected and recorded as 0 h, and then the leaf materials after spraying Me-JA and SA solution were collected at 4 h, 8 h, 12 h, 16 h, 20 h and 24 h, respectively.

### 4.2. Database Search and Sequence Analysis

The CTL amino acid sequences of *A. thaliana* (GCA_000001735.2, TAIR10.1), *S. lycopersicum* (GCA_000188115.3, SL3.0) and *O. sativa* (GCA_001433935.1, IRGSP-1.0) were collected from the National Center for Biotechnology Information (NCBI) database (https://www.ncbi.nlm.nih.gov/, accessed on 1 July 2020) and published literature [2,44]. Then used the above three species respectively, the information of petunia amino acid sequence were searched and collected by using the Basic Local Alignment Search Tool in the Solanaceae Genomic Database (https://solgenomics.net/, accessed on 3 July 2020). Meanwhile, the amino acid sequence information were also searched and collected using the word “chitinase” in the Petunia genome database (*Petunia axillaris* genome sequence v1.6.2). Subsequently, these sequence results obtained above were integrated and de-redundancy. And the conserved domains of the non-redundant sequences were retrieved using the CD-search function in NCBI to ensure the correctness.

### 4.3. Phylogenetic Analysis

The phylogenetic tree of five species (*P. axillaris*, *A. thaliana*, *Z. mays*, *P. abies*, and *H. sapiens*) was constructed by using the neighbor-joining (NJ) phylogenetic tree function of the MEGAX software (1000 bootstrap replicates) [45]. Then, we used EvolView online to beautify the phylogenetic tree (https://www.evolgenius.info, accessed on 8 July 2020) [46]. The phylogenetic relationships of *CTLs* in five Solanaceae species (*P. axillaris*, *N. tabacum*, *S. lycopersicum*, *S. tuberosum*, and *C. annuum*) and *A. thaliana* were constructed using the MUSCLE software online (http://www.drive5.com/muscle/, accessed on 8 July 2020) [47]. Phylogenetic groups were assigned according to the ortholog structure and functional domain published in previous research [2,12,26,27,28,29,30,31,32,48].

The phylogenetic relationship between *P. axillaris* and *A. thaliana* alone was also analyzed by using a NJ function in the MEGAX software (1000 bootstrap replicates). Using the method of Kim et al. [49] and lyu et al. [50], the extent of lineage-specific expansion of the CTL genes between the two species was investigated. The branches were defined by identifying nodes representing speciation events (dots). For those branches, a bootstrap value higher than 50% indicated the criteria for the least number of common ancestral *CTLs* between *P. axillaris* and *A. thaliana*. The branches containing only sequences for one of the two plants indicated that gene loss had occurred during evolution.

The homeology analysis map of *CTLs* among *P. axillaris*, *A. thaliana*, and *S. lycopersicum* genome was analyzed by TBtools software [51]. The genome sequence and annotation files of *A. thaliana*, *P. axillaris* and *S. lycopersicum* were downloaded from the NCBI database and the Solanaceae Genomics Network respectively. We selected the *PaCTLs* paralog pairs and homolog CTL genes between *S. lycopersicum* and *P. axillaris* with a similarity of over 80% to calculate the nonsynonymous rate (*Ka*), synonymous rate (*Ks*), and the *Ka*/*Ks* ratio using the TBtools software. 

### 4.4. Gene Structure, Conserved Motifs, and Cis-Acting Element Analyses

Signal peptide sequence prediction of the *PaCTLs* was performed using the SignalP-5.0 Server (http://www.cbs.dtu.dk/services/SignalP/, accessed on 9 July 2020) [52]. The exon-intron structure was obtained by aligning the coding sequence and corresponding genomic sequence using the TBtools software. The conserved motifs were recognized using the MEME tool version 5.1.1 (http://meme-suite.org/tools/meme, accessed on 12 July 2020) [53] with the maximum number of motifs set at 16. Pfam search were used online in the NCBI database.

We obtained the petunia genome model sequence in the Solanaceae genome network to analyze the cis-acting elements of *PaCTLs*. The 2-kb sequences upstream of the “ATG” were extracted and obtained using the TBtools software. Then, the obtained sequences were submitted to the PlantCare website (http://bioinformatics.psb.ugent.be/webtools/plantcare/html/, accessed on 15 July 2020) for the prediction of cis-acting elements [54]. The informations of the predicted cis-acting elements were visualized using the TBtools software.

### 4.5. Expression Analysis by qRT-PCR

Roots, stems, leaves, 2–3 cm flower buds, 4–5 cm flower buds, open flowers, and tender capsules collected from robust *P. axillaris* plants, and the seedlings treated with abiotic stress and the leaf material treated with hormones were collected for RNA extraction. The total RNA of the tender capsules were extracted using the RNAprep Pure Plant Kit (Polysaccharide and Polyphenol-rich, Tiangen Biotech, Beijing). Total RNA of other tissues were extracted by using the Trizol kit (TransGen Biotech, Beijing). The extraction procedure was performed according to the kit’s instructions. Then, the extracted total RNAs were detected using a 1.5% agarose gel to ensure their quality. First-strand cDNA was synthesized by using the PrimeScript RT Reagent Kit (TransGen Biotech, Beijing) in accordance with the manufacturer’s instruction.

qRT-PCR was used to examine the transcript levels of *PaCTLs*. Specific primers were designed using the primer design tool on the NCBI website (https://www.ncbi.nlm.nih.gov, accessed on 25 July 2020) based on multiple sequence alignment (Table S7). The constitutively expressed gene *PaUBQ* (F: 5′-GTGAATTATAGAATCGAGCATC-3′, R: 5′-AAATCAGAAACAATCCCAAC-3′) was selected as an internal control. qRT-PCR was performed using the CFX96 Real Time System (Bio-Rad, USA) machine. The PCR conditions were as follows: 30 s at 94 °C and followed by 42 cycles each of 5 s at 94 °C, 15 s at 60 °C, and 10 s at 72 °C. Each sample was run in triplicates with 15 μL of the reaction volume by using the TransStart Top Green qPCR SuperMix Kit (Transgen Biotech, Beijing, China). The 2^−ΔΔCt^ method was used to calculate the relative expression levels. The histograms were generated using GraphPad Prism 8 software.

### 4.6. Subcellular Localization

We selected four *PaCTLs* which had a higher expression level in flower organ development and stress environment, to investigate their subcellular localizations by generating C-terminal yellow fluorescent protein (YFP) fusions. Based on their CDS provided by the petunia database, we designed four specific primers (Table S8) for nucleotide fragment amplification. The PCR amplification reaction was carried out by using the high-fidelity enzyme 2×TransStart^®^ FastPfu PCR SuperMiX, in accordance with the manufacturer’s instruction. The resulting fragment was cloned into the fusion expression vector pB7YWG2–YFP under the control of the constitutive CaMV35S promoter, to create the fusion vector. Colonies containing the appropriate insert were identified by sequencing. The fusion vector was then transiently transformed into onion epidermal cells by particle bombardment. The fluorescence signal was analyzed after 16 h of incubation with the confocal laser microscope. The onion epidermal cells were plasmolyzed by infiltrating 0.3 g·mL^−1^ sucrose.

## 5. Conclusions

We identified 33 *PaCTL* gene family members from the genome of *P. axillaris*. The increase of GH18 subgroup members might be the primary driving force for their expansion. The *PaCTLs* were differentially expressed in vegetative and reproductive organs of petunia. Meanwhile, they showed varying degrees of response to abiotic stress (low temperature, high temperature, drought, and salinity stress) and hormone treatment (exogenous SA and Me-JA treatment), which meant that *PaCTLs* may have potential stress resistance functions. Furthermore, the subcellular localization result indicated that *PaCTLs* could localize on the cell wall to function. The present results provided a foundation for the exploration of *PaCTLs*’ function and for the functional identification of stress-resistance genes in petunia.

## Data Availability

All data in the present study are available in the public database as referred in the Section 4.

## Supplementary Materials

The following supporting information can be downloaded at: https://www.mdpi.com/article/10.3390/plants11091269/s1, **Figure S1**. Multiple sequence alignment of the GH18 sub-group *PaCTLs*. **Figure S2**. Multiple sequence alignment of the GH19 sub-group *PaCTLs*. **Figure S3**. A Neighbor-joining phylogenetic tree of CTL orthologs from five Solanaceae species (*P. axillaris*, *N. tabacum*, *S. lycopersicum*, *S. tuberosum*, *C. annuum*) and *A. thaliana*. **Table S1**. The *Chitinase-like gene* family members identified in *P. axillaris*. **Table S2**. Prediction of subcellular localization of the PaCTL proteins. **Table S3**. Calculation of substitution rates of homologues *CTLs* between *S. lycopersicum* and *P. axillaris*. **Table S4**. Number of *CTLs* in the five Solanaceae species and *A. thaliana* genomes. **Table S5**. The *Ka*/*Ks* analysis of *PaCTLs* gene pairs. **Table S6**. Statistical information of the gene expression of *PaCTLs* under normal and stress environment conditions. **Table S7**. The qRT-PCR primer for *PaCTLs*. **Table S8**. The amplification primer for *PaCTLs*.
