# Supplementary material for: Genome-Wide Identification and Expression Analysis of Chitinase-like Genes in Petunia axillaris
Source: Plants (Basel). 2022 May 9;11(9):1269. doi: 10.3390/plants11091269 (PMC9100346; doi:10.3390/plants11091269)
Supplement: Supplementary file 1 [file plants-11-01269-s001.zip › Figures S1¿CS3.pdf]

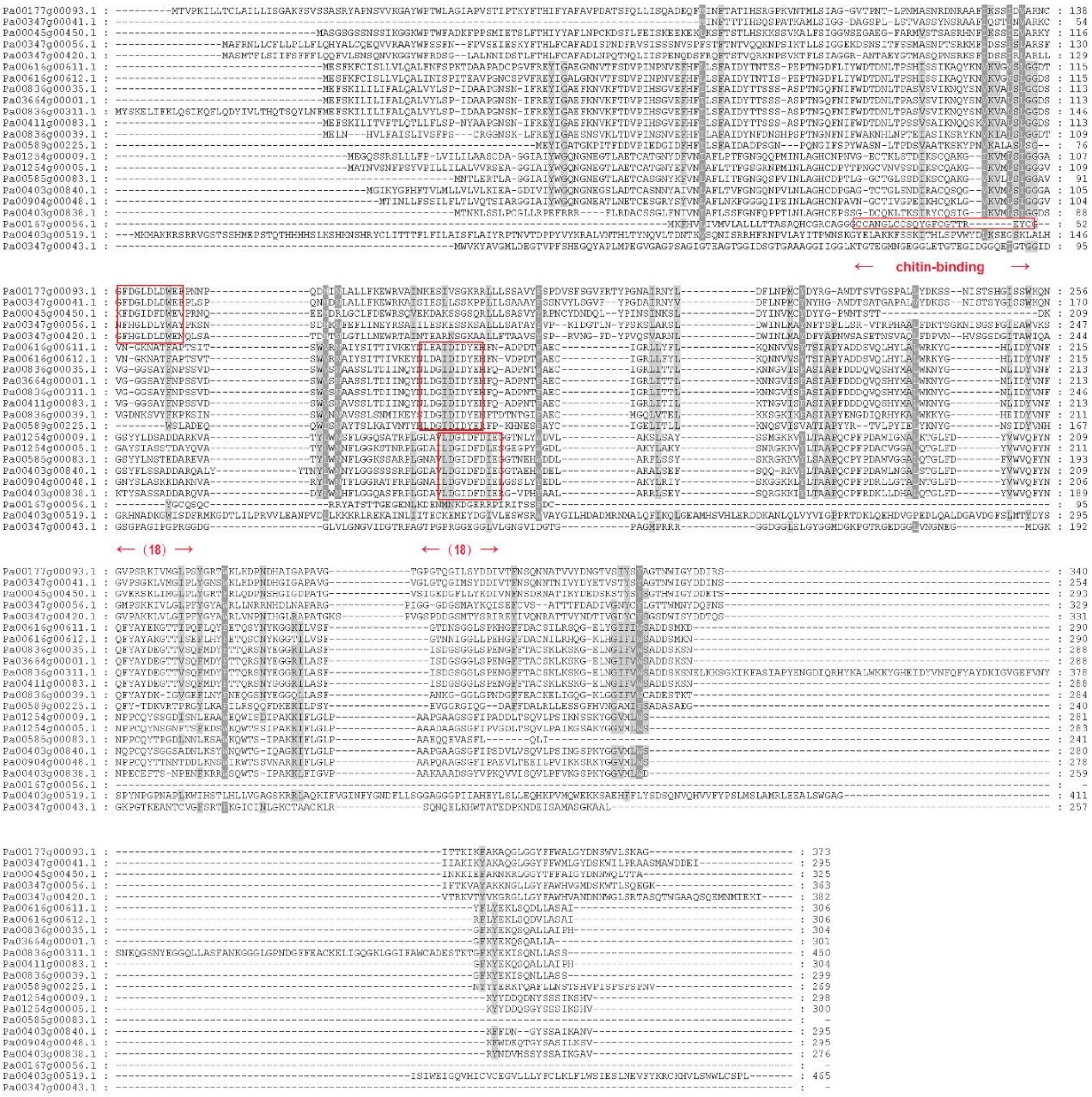

Figure S1. Multiple sequence alignment of the GH18 sub-group PaCTLs. The degree of shading shows the degree of similarity, and the common mode is displayed above the alignment sequence, with its name displayed below the sequence. (18) indicates the chitinase GH18 signal PS01095 ([LIVMFY]-[DN]-G-[LIVMF]-[DN]-[LIVMF]-[DN]-x-E), and Chitin-binding means chitin recognition or binding domain signal PS00026 (C-x(4,5)-C-C-S-x(2)-G-x-c-g-x(4)-[FYW]-C).

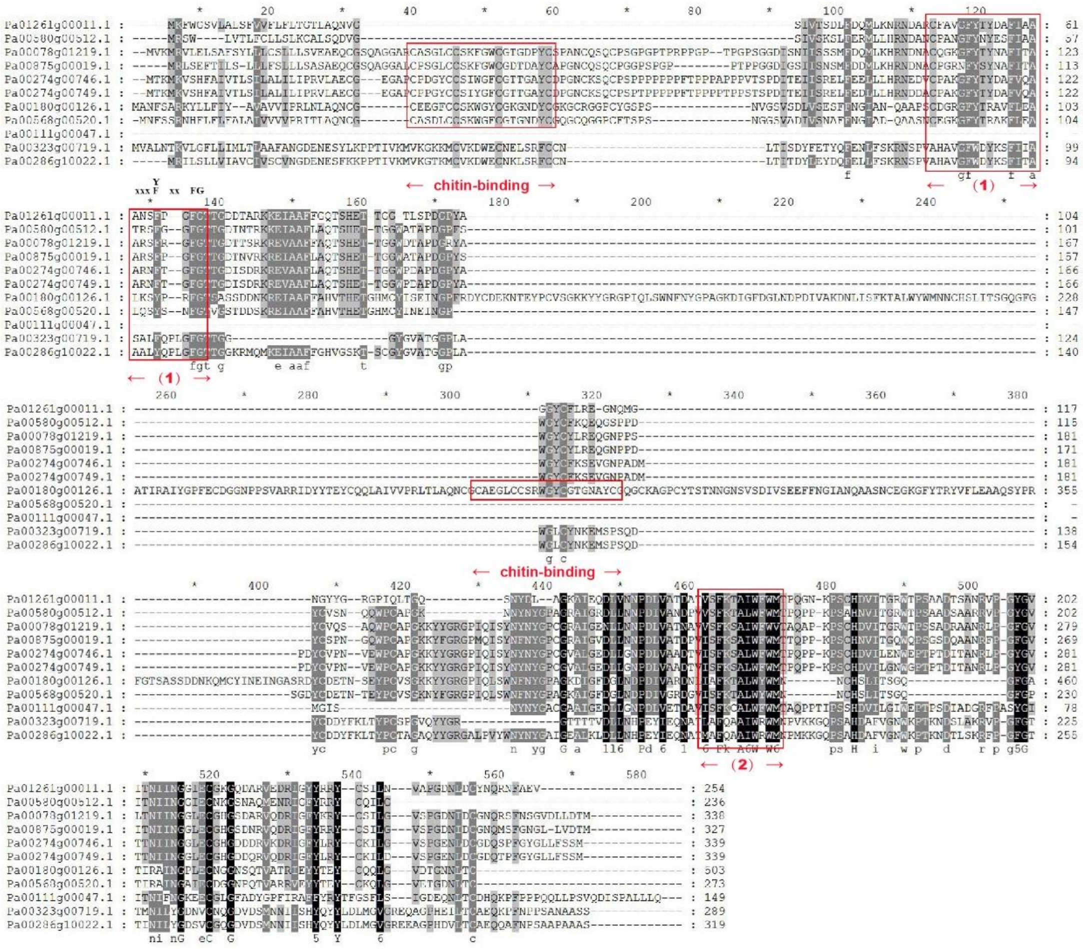

Figure S2. Multiple sequence alignment of the GH19 sub-group PaCTLs. The degree of shading shows the degree of similarity, and the common mode is displayed above the alignment sequence, with its name displayed below the sequence. (1) represents the chitinase GH19_1 signal PS00773 (Cx(4,5)-FY-[ST]-x(3)-[FY]-[LIVMF]-xAx(3)-[YF]-x(2)-F-[GSA]); (2) represents the chitinase GH19_2 signal PS00774 ([LIVM]-[GSA]-Fx-[STAG](2)-[LIVMFY]-W-[FY]-W-[LIVM]), and Chitin-binding means chitin recognition or binding domain signal PS00026 (C-x(4,5)-C-C-S-x(2)-G-x-c-g-x(4)-[FYW]-C).

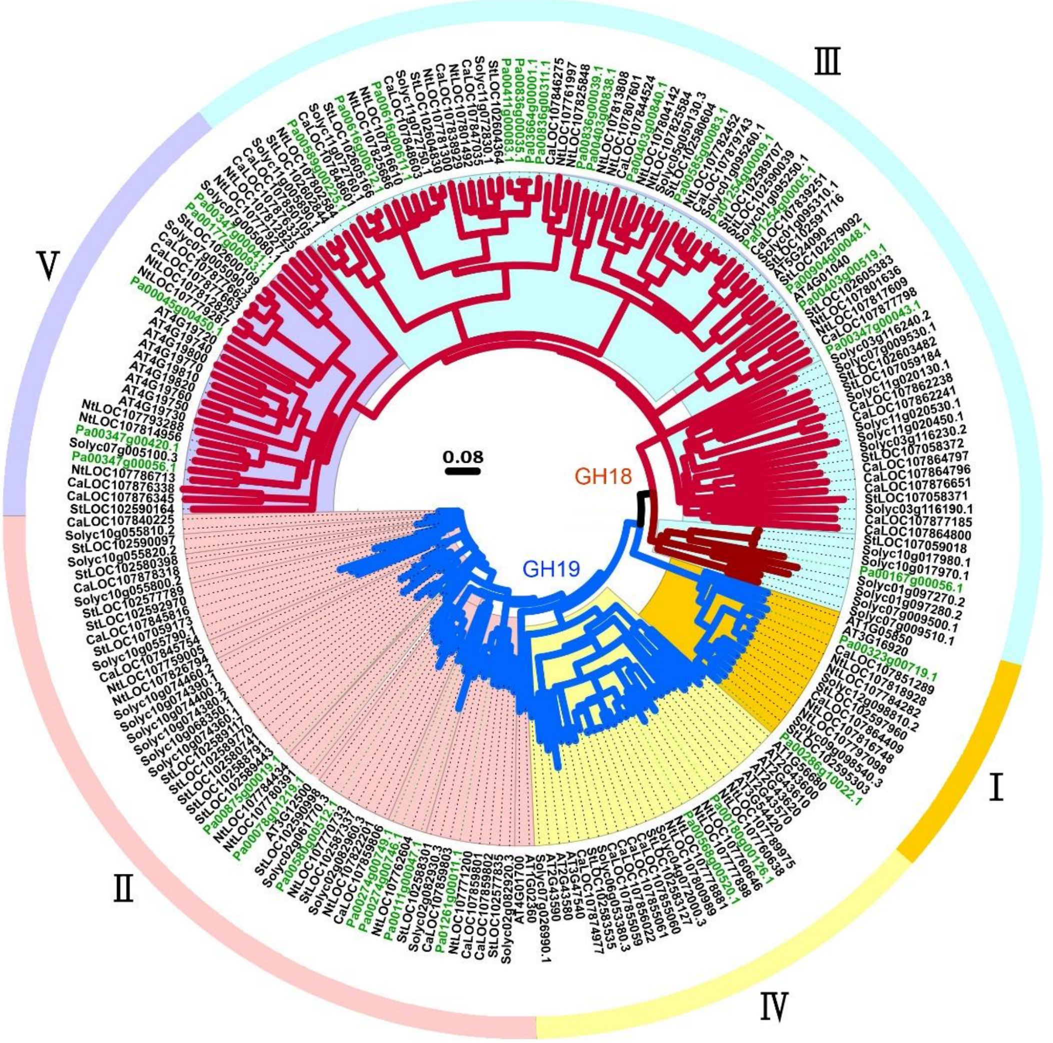

Figure S3. A Neighbor-joining phylogenetic tree of CTL orthologs from five *Solanaceae* species (*P. axillaris*, *N. tabacum*, *S. lycopersicum*, *S. tuberosum*, *C. annuum*) and *A. thaliana*.
